# The Phylogeography and Population Demography of the Yunnan Caecilian (*Ichthyophis bannanicus*): Massive Rivers as Barriers to Gene Flow

**DOI:** 10.1371/journal.pone.0125770

**Published:** 2015-04-27

**Authors:** Hui Wang, Xia Luo, Shaoquan Meng, Yongjian Bei, Tao Song, Tao Meng, Guifen Li, Baowei Zhang

**Affiliations:** 1 Anhui Key Laboratory of Eco-engineering and Bio-technique, School of Life Sciences, Anhui University, Hefei, 230601, Anhui, China; 2 College of Life Science & Technology, Yulin Normal University, Yulin, 537000, Guangxi, China; 3 Guangxi Forestry Inventory and Planning Institute, Nanning, 530011, Guangxi, China; University of Padova, ITALY

## Abstract

*Ichthyophis bannanicus* is the only caecilian species in China. In this study, the phylogeography and population demography of *I*. *bannanicus* were explored, based on the mitochondrial DNA genes (cyt *b* and ND2) and 15 polymorphic microsatellite loci. Altogether 158 individuals were collected from five populations in Yunnan province, Guangxi province, Guangdong province, and Northern Vietnam. Phylogeographical and population structure analysis identified either two groups (Xishuangbanna, Northern Vietnam-Yulin-Yangchun-Deqing) or three groups (Xishuangbanna, Northern Vietnam-Yulin-Yangchun, and Deqing), indicating that the Red River and Pearl River systems may have acted as gene-flow barriers for *I*. *bannanicus*. Historical population expansion that happened 15–17 Ka ago was detected for mtDNA data and was possibly triggered by warmer weather after the Last Glacial Maximum. However, the Bayesian simulations of population history based on microsatellite data pinpointed population decline in all populations since 19,123 to 1,029 years ago, demonstrating a significant influence of anthropogenic habitat alteration on *I*. *bannanicus*.

## Introduction

Caecilians (Gymnophiona or Apoda) are the least-known group of the three orders of living amphibian on account of their subterranean habits [1]. They are readily distinguished from frogs and salamanders by their worm-like, annulated bodies with tentacles and reduced eyes [2]. This group is found across much of the wet tropics and some subtropical regions apart from Madagascar, Australasia, and Southeast Asia east of Wallace’s line [2]. Caecilian taxonomy has been problematic even at the family level due to the lack of morphological and molecular data [3,4]. Currently, approximately 190 caecilian species in 10 families are recognized in Amphibiaweb [5] and the number is rapidly increasing due to rigorous regional surveys. In the face of global amphibian decline, more and more attention is being paid to amphibian species. However, compared with frogs and salamanders, little is known about caecilians. Among amphibian population genetic studies published between 2001 and 2010, about 68% of the studies were about frogs and 32% about salamanders, and caecilians were not represented at all [6].

The Yunnan caecilian (*Ichthyophis bannanicus*) is the only species of Gymnophiona in China. It was first found in the Yunnan Province in 1976 and initially identified as *Ichthyophis glutinosus* [7]. In 1984, based on more samples found in the Guangxi Province, it was classified as a new species and called *I*. *bannanicus* [8]. In 1985, it was also found in the Guangdong Province [9]. Since then, a few sporadic new occurrences have been reported in China, all restricted to the above three provinces. Recently, *I*. *bannanicus* was also found in Vietnam [10], Thailand and Laos [3]. Given the few records, the distribution of *I*. *bannanicus* is still unclear, and even less is known about its population size. The first survey of national terrestrial wild animals organized by the State Forestry Administration of China in 1995 reported that the population size of *I*. *bannanicus* was approximately 10,000 individuals (http://www.china.com.cn/chinese/PI-c/603534.htm). A pessimistic view was presented by the China Red Data Book of Endangered Animals, indicating that the number of *I*. *bannanicus* was only 400 and that it should be classified as an endangered species in China [11]. However, follow-up field surveys suggested that the population size was far bigger than the numbers proposed earlier. For example, an investigation on the population size of *I*. *bannanicus* in Beiliu City (Guangxi Province, China) showed approximately 10,800 individuals living in a single village in spite of the obvious population decline in this area [12]. In addition, Chou Wenhao reported to the Global Amphibian Assessment (GAA) workshop that about 200 individuals were collected in a single night in the Xishuangbanna National Reserve in 2000 [1]. Now, *I*. *bannanicus* is classified as near threatened (NT) in the China Species Red List [13]. All these data demonstrated that at least in some areas *I*. *bannanicus* still maintained a relatively large population size. However, little was known about the population genetics studies of the species needed to inform conservation planning.

It is well known that the genetic structure and population history of existing wildlife were profoundly affected by landscape features and/or historical climate fluctuations. In the range of *I*. *bannanicus*, well-developed river systems are one of the dominant landscape features, including the Yangtze, Pearl, Red, Mekong, Salween and Irrawaddy rivers and their criss-crossed tributaries. With typical amphibian features, the life history of *I*. *bannanicus* consists of an aquatic larval stage of about two years and a subterranean post-metamorphic stage in which the animals rarely enter the water [14,15]. Therefore, the distribution and phylogeographic patterns of *I*. *bannanicus* are thought to be influenced by river systems. Earlier studies indicated that *I*. *bannanicus* is strongly influenced by low temperatures, and when the temperature drops below 18°C, hibernation and even death may occur [15]. It is generally acknowledged that glaciers were never present in Southern China [16,17]. However, the fluctuating temperatures and water levels accompanying glaciations and deglaciations may have greatly affected the migration and dispersal of *I*. *bannanicus*. Nowadays, human activities are generally thought to be one of the major causes of habitat fragmentation and population declines for many endangered species [18–20]. Human impact may occur directly on species by hunting for instance, or indirectly by anthropogenic habitat changes. Human capture of caecilians was rarely reported due to their elusive behavior and lack of economic value. Therefore, anthropogenic habitat changes were regarded as the major cause of population decline or even local extinction for some caecilians [21,22]. On the other hand, certain agricultural practices such as crop plantations and fish farming are actually beneficial for caecilians, by providing suitable habitat (wet and loose soil), rather than having a negative impact. [23–26]. Although a trend of population decline was observed in field surveys [12], the abundance of *I*. *bannanicus* in synanthropic habitats (such as farmland) was higher than in primary habitats. However, whilst we know much more about how caecilians can survive or thrive in disturbed habitats, little is known about demographic changes due to primary habitat loss and adaptation to new habitat [1]. Therefore, it is necessary to estimate the impact of anthropogenic habitat changes on *I*. *bannanicus*.

In this study, we used multiple molecular markers including two mitochondrial DNA encoded genes, cytochrome *b* (cyt *b*) and NADH dehydrogenase 2 (ND2), and fifteen nuclear DNA microsatellite loci aiming: I. to evaluate population genetic diversity of *I*. *bannanicus*; II. to investigate the effects of landscape features and historical climate fluctuations on genetic structure and population evolutionary history; III. to assess the impacts of anthropogenic habitat changes on population demography.

## Materials and Methods

### Sample collection and DNA extraction

**Fig 1:**
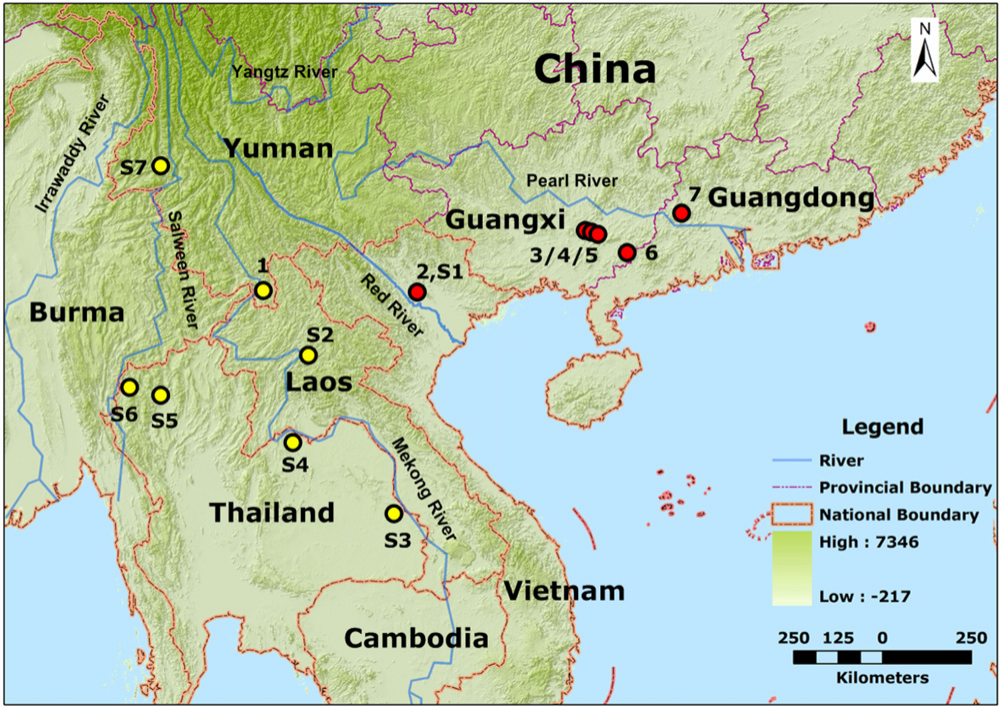
The study area and sampling localities of *I*. *bannanicus*. 1, Xishuangbanna, Yunnan province (BN); 2, Tam Dao, Vietnam (VN); 3–5, Yulin, Guangxi province (YL); 6, Yangchun, Guangxi province (YC); 7, Deqing, Guangdong province (DQ); S1, Tam Dao, Vietnam; S2, Laos; S3, Ubon Ratchathani Province, Thailand; S4, Phu Wan, Thailand; S5, Chiang Mai Province, Thailand; S6, Mae Hong Son, Thailand; S7, Longling, Yunnan, China.

One hundred and fifty-eight tissue samples of *I*. *bannanicus* were collected in southern China and northern Vietnam from 1998 to 2010 (Fig 1 and S1 Table). Samples were from seven localities in the five currently known distribution regions (defined here as populations): Xishuangbanna (BN, *N* = 34) from the Yunnan Province; Yulin (YL, *N* = 66) and Yangchun (YC, *N* = 19) from the Guangxi Province; Deqing (DQ, *N* = 25) from the Guangdong Province; Northern Vietnam (VN, *N* = 14) (S1 Table). Samples were preserved in 95% ethanol or stored at -20°C in the laboratory. Genomic DNA was isolated using the proteinase K digestion followed by SDS-phenol/chloroform method [27].

### Ethical statement

In the present study, collection of samples was performed within a long-term investigation project on *I*. *bannanicus* and all samples were from individuals that died naturally and were found during field work. This investigation project and the sample collection were approved by the Forestry Administration of Guangdong, Guangxi and Yunnan provinces. The specimen collection in Vietnam was conducted under the ethics license from the Vietnam National Museum of Nature, and was accepted by the Animal Research Ethics Committee of Anhui University and Yulin Normal University. Our experimental procedures complied with the current laws on animal welfare and research in China, and were specifically approved by the Animal Research Ethics Committee of Anhui University and Yulin Normal University.

### MtDNA sequencing and data analysis

Mitochondrial DNA fragments encoding cyt *b* and ND2 genes were amplified for 158 individuals. Amplification of the cyt *b* fragment was performed using the primers L14014 (5’-GGA TCT AAC CAA GAC TAA TGG TC-3’, forward) and H15198 (5’-GCT AAT GCT TTC TGA TAA GCT AC-3’, reverse) [28]. Amplification of the ND2 fragment was performed using the primers ND2-F (5’-CAC CTT CTA AGT AAG GTC AGC-3’) and ND2-R (5’-TCC TTC TTT GGG CTT TGA AGG-3’). All PCRs were performed with the same conditions in 50 μL: 20 to 80ng of genomic DNA, 25 μL 2×Easy*Taq* PCR SuperMix polymerase (TransGen Biotech, containing 1.25U Ex *Taq*, 0.4mM dNTP, 4mM Mg^2+^) and 0.4 μM of primers. Reactions were performed with the following profile: 5 min denaturing step at 95°C followed by 35 cycles of 30s at 95°C, 40s at 55°C and 40s at 72°C, and a final 10 min extension period at 72°C. PCR products were purified and subsequently sequenced on the ABI 3730 semi-automated Sequencer (PE Applied Biosystems). The cyt *b* DNA fragment was sequenced with the primers L14014 and L14500 (5’-GAG CCA CAG TAA TTA CAA ATC TC-3’), and ND2 was sequenced with the primers ND2-F and ND2-R. Nucleotide sequences were checked by eye and assembled using the program SeqMan in DNAStar [29], and open-reading frame for the two genes were verified. Cyt *b* and ND2 genes were combined together for analysis and aligned in MEGA 5.0 [30]. Nucleotide diversity (π, Nei 1987), haplotype diversity (*h*, Nei 1987) and haplotypes were identified using DnaSP v5.10.1 [31]. Genetic differentiation (*F*st) between populations was calculated as implemented in Arlequin 3.5.1.3 [32] based on haplotype frequency differences with 10,000 permutations. The best-fitting nucleotide substitution model for sequence data was identified using the software MrModeltest 2.3 [33] and the GTR+G substitution model was selected. We estimated phylogeographic relationships using Bayesian Inference on the combined cyt *b* and ND2 haplotype sequences, implemented in BEAST 1.7.4. [34]. The homologous sequence of *Ichthyophis glutinosus* (AY456251.1) was used as outgroup. Two independent runs of Markov Chain Monte Carlo (MCMC) analyses for 30 million generations were conducted with sampling every 1,000 generations, and 10% of the initial samples were discarded as “burn-in”. The convergence of chains was confirmed until average standard deviation of split frequency was below 0.01, and Bayesian Posterior Probabilities (BPP) were used as an indicator of node credibility (≥ 95% was considered significant) [35]. A median-joining network was constructed to depict relationships among haplotypes using Network 4.6.1.2 [36]. In addition, an independent phylogenetic analysis was repeated, combining the haplotypes found in the present study with seven published cyt *b* sequences of *I*. *bannanicus* (S1–S7, S1 from Northern Vietnam, S2 from Laos, S3–S6 from Thailand and S7 from Yunnan, China, Fig 1 and S1 Table) [3,37].

Historical demographic inferences were carried out using the Bayesian Skyline Plot (BSP) as implemented in the software BEAST with GTR+G substitution model. Two independent runs of Markov Chain Monte Carlo (MCMC) analyses for 30 million generations were conducted with sampling every 1,000 generations and 10% of the initial samples were discarded as “burn-in”. Additionally, to scale the time axis on BSPs, we used data estimates for the most recent common ancestor (MRCA) of all haplotypes in a lineage. Lacking fossil evidence, we assumed a range of substitution rate of 0.50–1.00% per Myr for cyt *b* or ND2 based on evolutionary rates commonly proposed for amphibians [38–43]. Plots for each analysis were visualized using Tracer 1.4 [44]. Tajima’s *D* [45] and *F*u’s *F*s [46] were calculated using Arlequin 3.5.1.3.

### Microsatellite genotyping and data analysis

Fifteen polymorphic tetranucleotide microsatellite loci for *I*. *bannanicus* were screened in the present study [47]. PCR reaction was performed in 20 μL system containing 20–50 ng of template DNA, 10 μL 2×Easy*Taq* PCR SuperMix polymerase (TransGen Biotech, containing 1.25U Ex *Taq*, 0.4mM dNTP, 4mM Mg^2+^) and 0.2 μM of each primer. Thermal cycling was performed with the following profile: 5 min denaturing step at 95°C; followed by 35 cycles of 30s at 95°C, 1 min at optimal annealing temperature [47] and 1 min at 72°C; and a final extension at 72°C for 10 min. All PCR products were visualized on an ABI 3730 semi-automated sequencer (PE Applied Biosystems) with GS500 marker and analyzed using GENEMARKER 1.85 (version 1.3, SoftGenetics LLC).

Micro-Checker [48] was used to detect the presence of null alleles and genotyping errors in microsatellite genotyping. Genetic variation was assessed by summary statistics, including mean number of alleles per locus (MNA), observed heterozygosities (*H*_O_), expected heterozygosities (*H*_E_) and inbreeding coefficients (*F*_IS_), which were performed for each population using GENETIX 4.02 [49]. Deviation from Hardy-Weinberg equilibrium (HWE) across all loci for each population was assessed using an exact probability test implemented in GENEPOP 4.2.1 [50]. Significance values for multiple comparisons were adjusted using the Bonferroni correction [51].

Genetic differentiation between populations was quantified using *F*_ST_ values which were calculated in GENETIX. In contrast to the preceding analyses (based on region-defined populations), a Bayesian clustering approach was used to estimate the number of potential clusters present in the microsatellite data and to assign individuals to inferred clusters using the STRUCTURE software [52]. The posterior probability for different values of *K* (the number of population) tested was set from 1 to 8, and ten independent runs were performed at the length of 1,000,000 MCMC with a 100,000 burn-in period using no prior information and assuming admixture and correlated allele frequencies. The true *K* was selected using the maximal value of the log likelihood [Ln Pr(*X*/*K*)] of the posterior probability of the date for a given *K* [52]. Further, the statistic Δ*K*, the second-order rate of change in the log probability of the data between successive values of *K* was also derived [53]. The results were graphically displayed by the software DISTRUCT [54].

Demographic history based on microsatellites was assessed using the following methods. First, the Wilcoxon’s sign rank test [55] was used to test for heterozygosity excess under the stepwise mutation model (SMM) and two-phase mutation model (TPM), with 95% single step mutations and 5% multi-step mutations and a variance of 12 following the recommendations of Piry et al. [56]. Second, a mode-shift test [57] was carried out to detect any distortion of the expected L-shaped distribution of allele frequency. Both Wilcoxon’s sign rank test and mode-shift test were performed in BOTTLENECK 1.2.02 [56]. Finally, we inferred past demographic change of populations from microsatellite data using MSVAR 1.3 [58] with a coalescent simulation-based Bayesian likelihood method. This approach employs coalescent MCMC simulations to estimate the posterior probability distribution of population parameters, based on the observed distribution of microsatellite alleles and their repeat numbers. The main parameters from MSVAR are: current effective population size (*N*_0_), historical or ancestral effective population size (*N*_1_) and the time since the beginning of population change (*T*). Five independent simulations were run on subsamples of five populations. In every simulation, we ran each chain with a thinning interval of 10,000 steps, leading to a total number of Monte Carlo searches of 1×10^9^ steps with the first 10% of total iterations discarded as burn-in. The remaining data were used to obtain the lower (5%), the median (50%), and the upper (95%) quantiles of the posterior distributions. Different means for the mean *N*_1_ were used to represent three demographic histories: a stable population (*N*_0_ = *N*_1_), an expanding population (*N*_0_>*N*_1_) and a decreasing population (*N*_0_<*N*_1_). The generation time of *I*. *bannanicus* was set to 5 years [59,60]. We estimated the marginal posterior distributions of the model parameters using the LOCFIT package [61] implemented in R v2.11.1 (R Development Core Team, 2010).

## Results

### Genetic diversity

A total of 2,181 base pairs of mtDNA (1038 for ND2 and 1,143 for cyt *b*) were obtained from 158 individuals after alignment. A total of 85 variable nucleotide sites were found with no insertions or deletions and 23 different haplotypes (H01-H23) were identified (S2 Table, GenBank accession numbers KM513544-KM513566). All haplotypes were restricted to a single population except for H08, which was the most common haplotype, and shared by four populations (VN/YL/YC/DQ) (S2 Table). Summary statistics, including haplotype richness, haplotype diversity, and nucleotide diversity within each population are given in Table 1. On the whole, a moderate genetic diversity was detected in *I*. *bannanicus* with an average haplotype diversity of *h* = 0.703 and a nucleotide diversity of *π* = 0.0077 (Table 1). The value of both haplotype diversity (*h* = 0.681) and nucleotide diversity (*π* = 0.0021) were remarkable higher for BN than the other populations (Table 1).

**Table 1:** Genetic variation and neutral test based on mitochondrial control region and fifteen microsatellite loci in five populations of *I*. *bannanicus*.

Population	*N*	*H*	*h* ± SD	π ± SD	MNA	*H*_O_	*H*_E_	*F*_IS_	Tajima’s *D* (*P*-value)	*F*u’s *F*s (*P*-value)
**BN**	34	7	0.681 ± 0.072	0.0021 ± 0.0012	8.29	0.666 ± 0.218	0.715 ± 0.0202	0.085(0.027–0.088)	-1.9478 (0.008)	2.9332 (0.895)
**VN**	14	1	0.000 ± 0.000	0.0000 ± 0.0000	3.64	0.608 ± 0.330	0.479 ± 0.255	-0.231(-0.303–0.215)	0.0000 (1.000)	-
**YL**	66	12	0.446 ± 0.076	0.0003 ± 0.0002	6.43	0.484 ± 0.207	0.515 ± 0.198	0.069(0.016–0.093)	-2.2733 (0.001)	-12.1574 (0.000)
**YC**	19	3	0.205 ± 0.119	0.0001 ± 0.0001	3.71	0.502 ± 0.230	0.490 ± 0.207	-0.019(-0.116–0.081)	-1.5108 (0.044)	-1.8044 (0.015)
**DQ**	25	3	0.157 ± 0.096	0.0001 ± 0.0001	5.29	0.462 ± 0.170	0.490 ± 0.193	0.077(-0.300–0.146)	-1.7333 (0.013)	-1.4037 (0.032
**Total**	158	23	0.703 ± 0.035	0.0077 ± 0.0038	12.43	0.533 ± 0.181	0.674 ± 0.135	0.213(0.190–0.235)	0.3478 (0.706)	9.2610 (0.961)

*N*, number of individuals; SD, standard deviation; *H*, haplotype number; *h*, haplotype diversity; π, nucleotide diversity; MNA, mean number of allele per locus; *H*_O_ and *H*_E_, observed and expected heterozygosity; *F*_IS_, inbreeding coefficient.

For microsatellites, 158 individuals were successfully genotyped at fifteen loci. Microsatellite genotyping raw data are given in S3 Table. Micro-Checker indicated no evidence of null alleles nor genotyping errors such as large allele dropout or stuttering. The MNA for the five populations ranged from 3.64 to 8.29 with a mean value of 12.43. Observed heterozygosity (*H*_O_ ± SD) ranged from 0.462 ± 0.17 to 0.666 ± 0.218, and expected heterozygosity (*H*_E_ ± SD) ranged from 0.479 ± 0.255 to 0.715 ± 0.202. In accordance with mitochondrial data, the BN population had the highest MNA, *H*_O_ and *H*_E_ (Table 1). There was no deviation in HWE apart from five loci in the VN population prior to Bonferroni correction. There was no linkage disequilibrium at any locus in any population.

### Phylogeography and divergence dating

**Fig 2:**
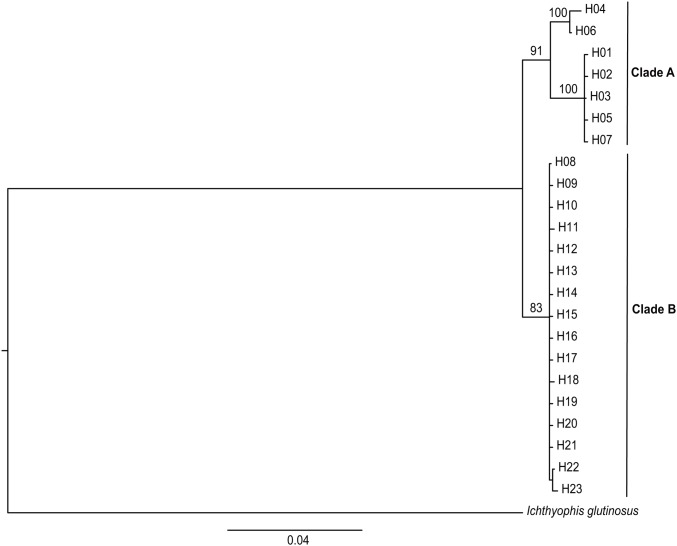
The Bayesian tree of 23 haplotypes from mitochondrial data of *I*. *bannanicus* with *I*. *glutinosus* as outgroup. Value above each branch refers to the Bayesian posterior probability (BPP).

The Bayesian tree revealed that there were two distinct haplotype groups with high bootstrap support: Clade A included seven haplotypes (H01–H07) all from the BN population, and Clade B included all the other sixteen haplotypes (H08–H23) (Fig 2). Within Clade A, H04 and H06 were divided from the other haplotypes and formed a distinct subclade. Clades A and B shared their most recent common ancestor (MRCA) about 1.39 million years before present (Ma BP) (95% HPD, 0.84–1.7 Ma). The phylogenetic tree of the above 23 haplotypes mixed with seven published cyt *b* sequences constructed with 714 homologous base pairs also showed two major lineages as above (S1 Fig). As for the seven new sequences, sample S1 from northern Vietnam joined Clade B and S2–S7 from Thailand and Laos merged into Clade A (S1 Fig).

**Fig 3:**
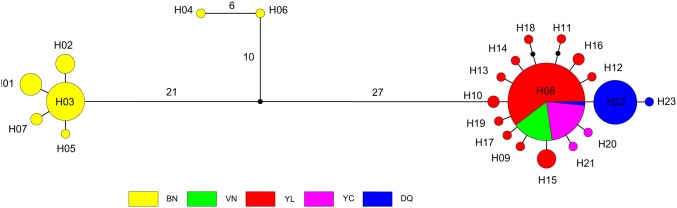
Median-joining network based on maximum parsimony among mtDNA haplotypes of *I*. *bannanicus*. Nodes contain the haplotype name and are proportional to haplotype frequencies. Length of branches is proportional to the number of changes from one haplotype to the following, with a number next to the branch representing more than one mutation step. Black nodes indicate undetected intermediate haplotype states.

The median-joining network showed a clear pattern with obvious phylogeographic structure in accordance with the Bayesian tree (Fig 3). Two haplogroups were distinguished from the network analysis: Clade A was composed of H01–H07, and Clade B contained H08–H23. Within Clade A, H04 and H06 were far from the other five closely linked haplotypes. In Clade B, haplotypes clustered together closely around dominant haplotype H08 and presented a typical star-like distribution. H22 was another dominant haplotype in group B, containing most individuals (23) from the DQ population (25 individuals overall).

### Genetic structure

**Fig 4:**
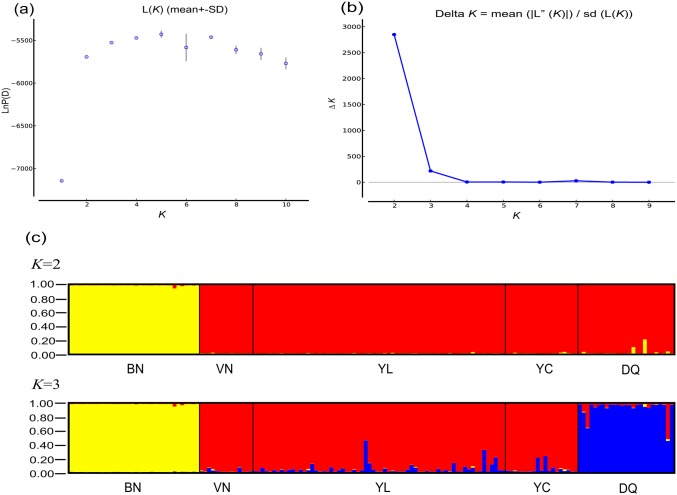
Bayesian STRUCTURE clustering results based on microsatellite genotypes among five *I*. *bannanicus* populations. (a) The linear relationship between LnP(D) and *K*, (b) Δ*K* values as a function of *K* based on 5 runs, (c) STRUCTURE output at *K* = 2 and 3.

Genetic differentiation analysis produced similar results for mtDNA and microsatellite data: significant *F*_ST_ values (range 0.4548–0.5690 for mtDNA data and 0.3097–0.3358 for microsatellite data) were detected between BN and the other populations, and between DQ and all the other populations as well (range 0.5569–0.8964 for mtDNA data and 0.0878–0.3332 for microsatellite data). The *F*_ST_ values between VN, YL and YC ranged 0.0078–0.0554 for mtDNA (non-significant) and 0.0282–0.0811 for microsatellites, indicating weak to moderate genetic differentiation (Table 2). Based on STRUCTURE analysis, although no obvious maximum log likelihood of posterior probability was found [Ln*P* (*X/K*) = −5325.2], the Δ*K* statistics output showed a clear maximum at *K* = 2 (Fig 4a and 4b). When *K* = 2, two clear clusters were identified: one cluster (Yellow) was composed of individuals from BN, and the other cluster (Red) of individuals from all the other populations (Fig 4c). The Δ*K* value based on the STRUCTURE output showed a large value for *K* = 3, too (Fig 4c). Correspondingly, a new independent cluster (Blue), composed of all individuals from the DQ population appeared, which may indicate possible substructuring within clade B (Fig 4c).

**Table 2:** Pairwise *F*_ST_ estimates based on mtDNA sequence (below diagonal) and microsatellite data (above diagonal), respectively.

Population	BN	VN	YL	YC	DQ
**BN**		0.3097***	0.3358***	0.3232***	0.3332***
**VN**	0.5690***		0.0627***	0.0811***	0.1715***
**YL**	0.4548***	0.0554		0.0282***	0.0878***
**YC**	0.5182***	0.0078	0.0143		0.1057***
**DQ**	0.5569***	0.8964***	0.6438***	0.8156***	

The significance was indicated after the Bonferroni correction:

*** *P*<0.001.

### Demography

**Fig 5:**
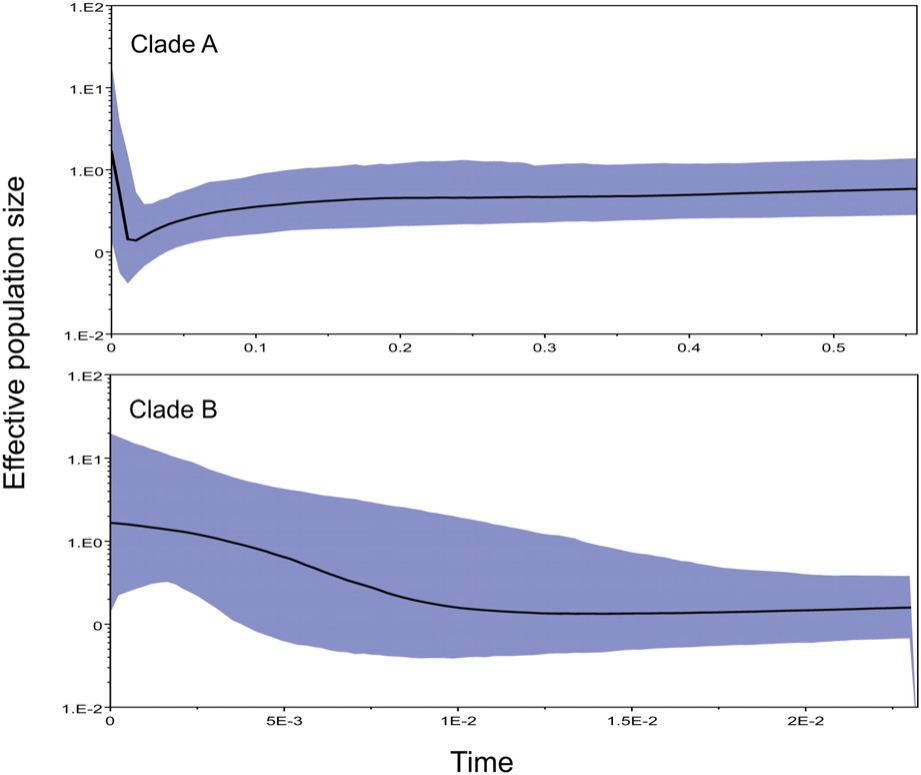
Bayesian skyline plot of effective population size. Time is expressed in million years.

Demographic analysis was carried out in five populations and in the two clades recovered by phylogenetic analysis (A and B). Significant negative Fu’s *F*s test and Tajima’s *D* test values were detected in YL, YC and DQ populations, supporting population expansion (Table 1). For BN population (equivalent to Clade A), Tajima’s *D* test provided a significant negative value, but Fu’s *F*s test showed conflicting results. Bayesian skyline plots (BSP) indicated that *I*. *bannanicus* maintained a relatively constant population size for quite a long time before a gradual decrease around 0.2 Ma BP, and then a strong growth occurred since approximate 16 thousand years before present (Ka BP) for Clade A (Fig 5). In contrast, Clade B maintained a constant population size before a strong growth approximately 14 Ka BP (Fig 5).

**Fig 6:**
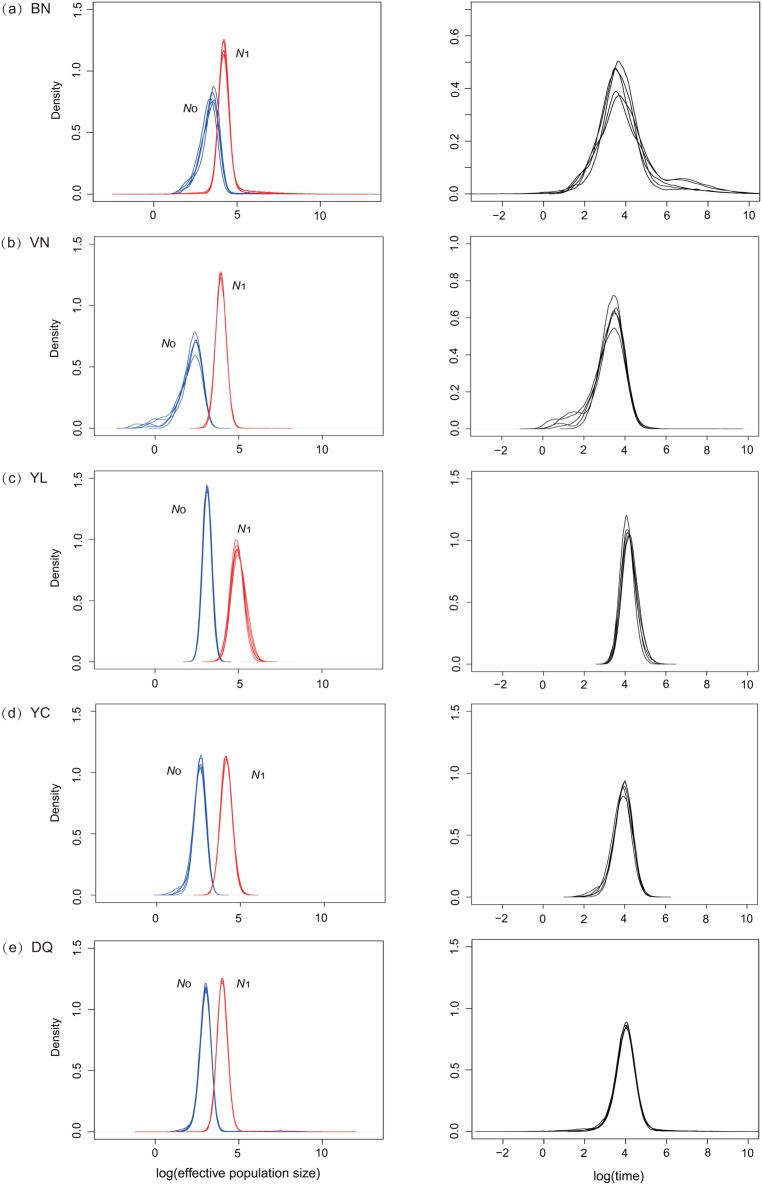
Estimated posterior distributions of current (*N*_0_, blue curve) and ancestral (*N*_1_, red curve) effective population sizes and time since population change (*T*, black curve) using MSVAR on a logarithmic scale based on a generation time of 5 years from five populations. (a) BN, (b) VN, (c) YL, (d) YC and (e) DQ.

Based on microsatellite data, BOTTLENECK indicated no significant signs of a recent bottleneck in all populations under both TPM and SMM. In addition, the mode-shift test showed a normal L-shaped distribution of allele frequencies in all populations. However, Bayesian coalescent simulation provided compelling evidence for population decline (Table 3, Fig 6). Summary statistics for the posterior distributions of *N*_0_, *N*_1_ and *T* were generated using MSVAR 1.3 with five independent replicates showing concordant results in all populations (Table 3, Fig 6). The BN population had the highest *N*_0_ (20366) and the YL population had the highest *N*_1_ (65574) while the VN population had the lowest *N*_0_ and *N*_1_ (170 and 8615). Overall, decline severity was low (*N*_0_/*N*_1_ ≥0.1) for BN, meanwhile moderate or severe population declines (*N*_0_/*N*_1_ ≥0.01) were detected in the VN, YL, YC and DQ populations. The medians of *T* for the five populations were 1029 (BN), 2140 (VN), 19123 (YL), 8182 (YC) and 9432 (DQ) respectively (Table 3, Fig 6).

**Table 3:** Medians of current (*N*_0_) and ancestral effective population sizes (*N*_1_) and time since population decline (*T*) for five populations based on a generation time of 5 years, using Bayesian MSVAR simulations.

Population	*N*_0_ ± SD	*N*_1_ ± SD	*T* (years) ± SD
**BN**	3889 ± 1058 (185–14 139)	20 366 ± 8139 (4376–137 500)	1029 ± 458 (126–10 967 134)
**VN**	170 ± 25 (4–1079)	8615 ± 170 (2528–29 406)	2 140 ± 346 (51–18 569)
**YL**	1 466 ± 530 (78–22 379)	65 574 ± 5265 (1400–7 873 575)	19123 ± 1617 (499–900 015)
**YC**	371 ± 24 (62–1385)	14 692 ± 458 (3752–57 962)	8 182 ± 793 (954–43 221)
**DQ**	989 ± 72 (219–3501)	10 319 ± 612 (3159–35 988)	9 432 ± 766 (1074–59 364)

Standard deviation (SD) was computed across at least five repeated runs, and values in the bracket were the 0.05 and 0.95 quantiles.

## Discussion

### Genetic diversity

In the present study, genetic diversity of *I*. *bannanicus* was analyzed based on mtDNA and microsatellite data. Average, mtDNA haplotype diversity (*h* = 0.703) was higher than in some extremely endangered amphibians, such as *Ranodon sibiricus* (*h* = 0.367) [62], and lower or close to some non-endangered species, such as *Rana nigromaculata* (*h* = 0.4046–0.9454) [63], and *Feirana quadranus* (*h* = 0.6599–0.9498) [64]. Based on microsatellite data, the average MNA and *H*_E_ were 12.43 and 0.674, respectively, suggesting high genetic diversity in *I*. *bannanicus*. Among the five studied populations, BN showed the highest genetic diversity based on either mtDNA or microsatellite data. The high ancestral effective population size *N*_1_ and low population decline severity of BN (Table 3, Fig 6) may explain its higher genetic diversity, and may be due to its larger ancestral population and longer population stablity.

### Gene flow barriers

Up to now, little is known about gene flow barriers or dispersal pattern for caecilian species. In the present study, deep genetic divergences and remarkable differentiations were detected among the five populations (Table 2, Fig 4). These results may point to possible gene flow barriers for *I*. *bannanicus*. The geographic distance between DQ population and YL/YC population (191 km) is much less than the distance between YL/YC and VN population (530 km). However, a remarkable differentiation was detected between DQ and YL/YC/VN but not within YL/YC/VN. Considering that there is neither obvious elevation difference nor other geographic barriers between DQ and its adjacent populations except the Pearl River, we inferred that the Pearl River may constitute an effective geographic barrier between DQ and the other populations. Generally speaking, the effectiveness of rivers as barriers to gene flow is obvious in small mammals [65,66]. But for amphibians, the barrier effect of rivers is uncertain. For example, Li *et al*. found major rivers to be barriers for the alpine stream frog *Scutiger boulengeri* [67], and the Chagres River was shown to be a barrier for the túngara frog *Physalaemus pustulosus* [68]. In contrast, other studies have shown that mountain ridges rather than rivers constituted genetic barriers for amphibians [69,70]. Well-developed river systems are one of the dominant landscape features throughout the range of *I*. *bannanicus*, including Yangtze River, Pearl River, Red river, Mekong River, Salween River, Irrawaddy River and their crisscrossed tributaries. It seems that the Red River may act as an effective gene flow barrier between BN and other populations in this study. Because of insufficient data, we do not know whether other massive rivers such as the Mekong River or Salween River are also acting as gene flow barriers.

The conclusion that massive rivers may act as gene flow barriers was in conflict with our original hypothesis. We originally thought that the optimal migration path for caecilian species was through drainage systems because of weak movement ability in subterranean life stage [28]. It would appear that crossing a large river is very difficult for *I*. *bannanicus*.

However, the genetic barrier effect of rivers is more unstable than that of mountain ridges due to changing hydrological factors like water level, width, flow rate, bed height and channel position which directly affect the effectiveness of the barrier. For example, the barrier effect on the plateau wood frog *Rana kukunoris* was much stronger at the downstream of the Yalong River, where the water flow was higher and the river valley steeper and drier [71]. During historical periods, these hydrological factors, especially water levels, fluctuated with climate fluctuation and so were obviously affected by the Quaternary glaciations in the Pleistocene. In the glacial periods, temperatures lowered and massive sheets of ice locked water, resulting in low water levels in river systems, weakening the barrier effect. In China during glacial periods, the mean annual temperatures dropped by about 10–12°C, the sea level fell by about 140 m and the paleo-coastline pushed about 600 km away from the present coastline [16,17], and the water level of the Yangtze River fell by about 20–45 m during the Last Glacial Maximum [72]. During interglacial periods, the temperature rose and more water entered the rivers, resulting in wider and larger flowing rivers that strengthened their barrier effect. In the present study, the divergences between Clades A and B were estimated to have occurred about 1.39 Ma BP, corresponding to the Donau—Günz Interglacial that occurred 1.7–1.3 Ma BP [73] which supported the proposed hypothesis viewing the Red River as the main geographic barrier between the two *I*. *bannanicus* clades.

### Demographic history

A strong historical population expansion signal was detected in both Clade A and B approximately 16 and 14 Ka BP respectively (Fig 5), which was just after the Last Glacial Maximum (LGM, 18–22 Ka BP). This post-LGM expansion event is not coincident with those of many Chinese species whose expansion events happened earlier than the LGM (e.g. [74–79]), but consistent with species in North America and Europe (e.g. [80–83]). We thought that this discordance was not about the geography or climate differences between *I*. *bannanicus* and other Chinese species. It may because that the *I*. *bannanicus* is more sensitive to low temperature than the other species [15]. Hence, we considered that the warmer weather after LGM might have triggered this rapid population expansion.

Although BOTTLENECK and the model-shift test did not support population declines, the MSVAR results proved strong population declines in all populations (Table 3, Fig 6). This inconsistent results can be ascribed to poorer ability of detecting neither too weak nor too recent population declines in BOTTLENECK than in MSVAR [84]. The times since population decline (*T*) were from 1,029 to 19, 123 years ago (Table 3, Fig 6), throughout the whole Neolithic Era when farming began. In southern China, rice cultivation appeared 10–12 Ka BP according to the fossil record [85,86]. Overall, the recent population declines of *I*. *bannanicus* could be attributed to human agricultural activities in the Neolithic Era. Farming in the Neolithic Era was mainly slash-and-burn cultivation which still survives in some minorities in southern China. Compared to modern farming, slash-and-burn had more severe impacts on the natural environment, and is still considered as an important threat in many areas nowadays [87]. Slash-and-burn practice and the resulting deforestation would have had various negative impacts on caecilians: first, individuals were swept away by the felling and fire because of their weak migration ability; second, because of poor soil fertility in slash-and-burned lands, the soil dwelling animals that the caecilians feed on would also decline [25,88]; third, because of the low soil fertility resulting from slash-and-burn agriculture, lands were abandoned after only a few years and new lands exploited, so spreading the negative impact. This land use pattern caused persistent and high-intensity habitat destruction for caecilians, thus explaining the population decline evidenced by MSVAR. For the BN population, the decline started later and was less severe than for the other populations (Table 3, Fig 6). Accordingly, in the early Holocene the whole of southern China was covered by evergreen broad-leaf forest [89]. However, at present, primeval forests remain only in Xishuangbanna (BN). We therefore suggest that these differences in the time and severity of decline are attributable to the less severe deforestation impact in Xishuangbanna than in other areas. Our data therefore strongly suggest that anthropogenic habitat alteration and deforestation led to *I*. *bannanicus* population declines with decline severity directly correlated to the degree of primary habitat destruction.

Fortunately, there is a view that current agricultural practices are not necessarily harmful to caecilians and may even be beneficial to them in some cases [23–26]. Throughout the range of *I*. *bannanicus*, the staple crop is rice and mechanization is relatively low. The moist and loose soil in paddy fields may benefit *I*. *bannanicus*. In addition, some researchers reported that caecilians can tolerate agrochemicals [1,25]. Therefore, the current success of *I*. *bannanicus* in the agricultural environment may be just a remarkable recovery from historical population declines.

## Supporting Information

S1 Fig The Bayesian tree of 23 haplotypes from mitochondrial data combined with seven published cyt *b* sequences of *I*. *bannanicus* with *I*. *glutinosus* as outgroup. Value above each branch refers to the Bayesian posterior probability (BPP).
(EPS)

S1 Table Samples used for analysis in this study together with the information on localities, coordinates, GenBank accession numbers for published sequences, populations and sample size. (DOC)

S2 Table Twenty-three haplotypes identified in this study with information on numbers of individual (N) and sample numbers. (DOC)

S3 Table Microsatellite genotyping raw data of fifteen loci. Abbreviations are the same as those in Fig 1. (XLS)
