# Supplementary material for: The Phylogeography and Population Demography of the Yunnan Caecilian (Ichthyophis bannanicus): Massive Rivers as Barriers to Gene Flow
Source: PLoS One. 2015 Apr 27;10(4):e0125770. doi: 10.1371/journal.pone.0125770 (PMC4411157; doi:10.1371/journal.pone.0125770)
Supplement: S1 Table — (DOC) [file pone.0125770.s002.doc]

**Table S1. Samples used for analysis in this study together with the information on localities, coordinates, GenBank accession numbers for published sequences, populations and sample size**

| **Locality no.** | **Locality** | **Coordinate** | **GenBank accession no.** | **Population** | **Sample size** |
| --- | --- | --- | --- | --- | --- |
| **1** | Mengla, Xishuangbanna, Yunnan Province, China | E101°26′, N21°28′ |  | BN | 34 |
| **2** | Tam Dao, Vietnam | E105°53′, N21°01′ |  | VN | 14 |
| **3** | Dawang, Yulin, Guangxi Province, China | E110°20′, N22°28′ |  | YL | 7 |
| **4** | Liuma, Yulin, Guangxi Province, China | E110°36′, N22°17′ |  | YL | 19 |
| **5** | Pingzheng, Yulin, Guangxi Province, China | E110°36′, N22°17′ |  | YL | 40 |
| **6** | Yangchun, Yulin, Guangxi Province, China | E110°52′, N22°09′ |  | YC | 19 |
| **7** | Deqing, Guangdong Province, China | E111°48′, N23°17′ |  | DQ | 25 |
| **S1** | Tam Dao, Vietnam |  | AY101259 |  |  |
| **S2** | Laos |  | AB686073 |  |  |
| **S3** | Ban Na Sabaeng, Ubon Ratchathani Province, Thailand |  | AY101260 |  |  |
| **S4** | Phu Wan, Thailand |  | AB686053 |  |  |
| **S5** | Mae Saivalley, Chiang Mai Province, Thailand |  | AY101261 |  |  |
| **S6** | Mae Hong Son, Thailand |  | AB686045 |  |  |
| **S7** | Longling, Yunnan, China |  | AY101255 |  |  |
