# Supplementary material for: The Phylogeography and Population Demography of the Yunnan Caecilian (Ichthyophis bannanicus): Massive Rivers as Barriers to Gene Flow
Source: PLoS One. 2015 Apr 27;10(4):e0125770. doi: 10.1371/journal.pone.0125770 (PMC4411157; doi:10.1371/journal.pone.0125770)
Supplement: S2 Table — (DOC) [file pone.0125770.s003.doc]

**Table S2.** Twenty-three haplotypes identified in this study with information on numbers of individual (N) and sample numbers

| **Haplotype** | **N** | **Sample no.** |
| --- | --- | --- |
| **H01** | 6 | BN01, BN11, BN15, BN17, BN23, BN27 |
| **H02** | 5 | BN02, BN12, BN21, BN28, BN30 |
| **H03** | 18 | BN03, BN04, BN06, BN08, BN09, BN13, BN14, BN16, BN18, BN19, BN20, BN22, BN25, BN26, BN29, BN31, BN32, BN34 |
| **H04** | 1 | BN05 |
| **H05** | 1 | BN07 |
| **H06** | 1 | BN10 |
| **H07** | 2 | BN24, BN33 |
| **H08** | 81 | VN01, VN02, VN03, VN04, VN05, VN06, VN07, VN08, VN09, VN10, VN11, VN12, VN13, VN14, YL93, YL94, YL95, YL96, YL98, YL99, YL01, YL05, YL06, YL07, YL08, YL09, YL11, YL12, YL13, YL15, YL16, YL17, YL18, YL19, YL33, YL35, YL37, YL38, YL39, YL40, YL41, YL42, YL44, YL45, YL46, YL47, YL48, YL49, YL50, YL52, YL53, YL55, YL56, YL57, YL61, YL62, YL64, YL66, YL67, YL100, YL101, YL102, YL103, YC20, YC22, YC23, YC24, YC25, YC26, YC27, YC29, YC30, YC31, YC32, YC105, YC106, YC107, YC108, YC109, YC110, DQ92 |
| **H09** | 1 | YL97 |
| **H10** | 2 | YL02, YL03 |
| **H11** | 1 | YL04 |
| **H12** | 1 | YL10 |
| **H13** | 1 | YL14 |
| **H14** | 1 | YL34 |
| **H15** | 5 | YL36, YL51, YL54, YL60, YL65 |
| **H16** | 2 | YL43, YL58 |
| **H17** | 1 | YL59 |
| **H18** | 1 | YL63 |
| **H19** | 1 | YL104 |
| **H20** | 1 | YC21 |
| **H21** | 1 | YC28 |
| **H22** | 23 | DQ68, DQ69, DQ70, DQ71, DQ72, DQ73, DQ74, DQ75, DQ76, DQ77, DQ78, DQ80, DQ81, DQ82, DQ83, DQ84, DQ85, DQ86, DQ87, DQ88, DQ89, DQ90, DQ91 |
| **H23** | 1 | DQ79 |
